# 3D Gas Sensing with Multiple Nano Aerial Vehicles: Interference Analysis, Algorithms and Experimental Validation †

**DOI:** 10.3390/s23208512

**Published:** 2023-10-17

**Authors:** Chiara Ercolani, Wanting Jin, Alcherio Martinoli

**Affiliations:** Distributed Intelligent Systems and Algorithms Laboratory, School of Architecture, Civil and Environmental Engineering, École Polytechnique Fédérale de Lausanne (EPFL), 1015 Lausanne, Switzerland; wanting.jin@epfl.ch (W.J.); alcherio.martinoli@epfl.ch (A.M.)

**Keywords:** gas sensing, multi-robot systems, informative path planning

## Abstract

Within the scope of the ongoing efforts to fight climate change, the application of multi-robot systems to environmental mapping and monitoring missions is a prominent approach aimed at increasing exploration efficiency. However, the application of such systems to gas sensing missions has yet to be extensively explored and presents some unique challenges, mainly due to the hard-to-sense and expensive-to-model nature of gas dispersion. For this paper, we explored the application of a multi-robot system composed of rotary-winged nano aerial vehicles to a gas sensing mission. We qualitatively and quantitatively analyzed the interference between different robots and the effect on their sensing performance. We then assessed this effect, by deploying several algorithms for 3D gas sensing with increasing levels of coordination in a state-of-the-art wind tunnel facility. The results show that multi-robot gas sensing missions can be robust against documented interference and degradation in their sensing performance. We additionally highlight the competitiveness of multi-robot strategies in gas source location performance with tight mission time constraints.

## 1. Introduction

The rise of extreme environmental phenomena in recent decades makes for a compelling case for carefully designing and planning environmental mapping and monitoring missions. To this end, robotic assets can be employed, to make such missions more efficient and less dangerous. The application of robotic platforms to environmental missions spans several domains. Some rely on images as input data, such as the mapping of weeds for agriculture [1]; others rely on scarcer data, such as the mapping of chemical components in water [2] or air [3]. Robotic Gas Distribution Mapping (GDM) missions aim to create a reliable map of gas distribution in a given environment, while Gas Source Localization (GSL) methods aim to provide an accurate guess of the gas leak location. The success of the GDM and GSL methods is tightly dependent on the constraints of the robotic platform used, such as time budget or versatility of motion. Bespoke navigation methods in these domains aim to improve the outcome of GDM and GSL algorithms, by focusing on areas of interest, often by employing adaptive sampling techniques. The application of a Multi-Robot System (MRS) to these domains also offers an interesting direction of research, aimed at improving the information gathering procedure in time-sensitive applications. Ideal platforms on which to perform gas detection tasks in indoor or GNSS-denied spaces are Nano Aerial Vehicles (NAVs). Their movement range can capture the tridimensionality of the gas dispersion, and their propellers cause a smaller perturbation compared to bigger drones, preserving their sensing capabilities [4,5,6]. However, the impact that an MRS mission composed of several vehicles with propellers can have on gas distribution has not been studied in the literature. For this paper, we explored multi-robot adaptive sampling techniques for GDM and GSL, focusing on studying the interference between different flying robotic agents and understanding its impact, experimentally.

### 1.1. Gas Distribution Mapping and Source Localization

GDM approaches aim to generate a map of the gas distribution of a given environment. They are usually composed of an estimation and a navigation part. The Kernel DM+V method is one of the most prominent estimation approaches in this domain [7]. It uses a Gaussian kernel to interpolate the sensed gas values and outputs the mean and variance of the gas distribution. Through the kernel interpolation, the algorithm is able to infer the value of the gas distribution in a volume of a few tens of centimeters around the measurement location. The method was extended to 3D in [8], and was successfully employed for gas mapping missions with one flying vehicle in 2D [9,10] and in 3D [4]. It must be noted that most of the work on estimation for GDM exploits locally interpolated data, instead of opting for model-based solutions. Non-adaptive navigation strategies, such as lawnmower movement, have been frequently employed in GDM missions. They mainly consist of preplanned trajectories with lengthy stops at measurement locations [5,9]. These trajectories are easy to generate, but can lead to coarse scans of the concerned volume when the time budget of the mission is limited. Adaptive navigation strategies have been explored in recent years, with a focus on Informative Path Planning (IPP). In IPP algorithms, informative quantities, such as entropy or mutual information, are used to steer the movement of the robots towards areas rich in information content. There are several examples of such techniques being employed in environmental monitoring missions [11], such as temperature field estimation [12] or weed monitoring [1]. In the context of gas mapping missions, characterized by a stochastic and highly dynamic underlying phenomenon, employing IPP strategies for navigation is quite challenging. Additionally, NAVs can disrupt the gas plume with the wake of their propellers, providing less precise information to the IPP algorithm. The amount of information that can be collected during a mission is also impacted often by flight time constraints. Nonetheless, IPP strategies have been successfully applied to GDM tasks, providing improved performance with respect to preplanned trajectories [4,10,13].

GSL algorithms aim to correctly locate the source of a gas leak. In recent years, most of the research in this domain has focused on probabilistic algorithms. Such algorithms model the belief about the location of the source of the gas leak, using a probability distribution. Each new observation prompts the algorithm to update the belief, using Bayesian estimation, until the probability distribution can be described by a Dirac distribution. The uncertainty associated with the estimation can be used to measure the quality of the source guess. A major advantage of using this class of algorithms, compared to other techniques, such as bio-inspired algorithms, is their ability to estimate several additional parameters of the source, such as the gas release rate [14]. Additionally, these algorithms offer remarkable versatility for deployment on a wide variety of sensing nodes endowed with varying degrees of mobility and with several single- and multi-node configurations. However, the high computational cost of these algorithms can make onboard deployment challenging on nodes with limited computational resources. The most notable probabilistic algorithms for GSL are Infotaxis [15] and Source-Term Estimation (STE) [16]. Within the scope of this work, we deployed GaSLAM [17], a method that combines GDM and GSL, on an MRS. The GSL part of this algorithm is based on the work presented in [18], which used STE for GSL.

### 1.2. Multi-Robot Systems for Gas Sensing

The performance of environmental mapping missions can be enhanced by the employment of an MRS coupled with IPP. More information about the phenomenon of interest can be gathered in a given time frame, the robustness of the system is increased—both with respect to hardware failures and sensing redundancy—and a mission can be conducted in less time or can achieve larger coverage. Moreover, a deeper understanding of a dynamic phenomenon can be achieved, by gathering several samples at different locations simultaneously. The majority of the approaches in this field are concerned with obtaining exhaustive coverage of the concerned volume [19]. Within the scope of this work, we considered that our mission was time sensitive. Therefore, while we wanted to cover as much volume as possible, we wanted the robots to focus on informative areas.

Mapping approaches for MRSs often couple adaptive sampling strategies with additional features, to improve the mapping outcome. Several approaches allocate optimally a set of predetermined locations to the robotic team, usually based on information content and location [20]. Other approaches exploit prior knowledge of the phenomenon, to make informative decisions [21], or they rely on a large amount of data, often coming from cameras [22,23]. Moreover, global knowledge can be injected into the planning, in the form of attractor landmarks [22] or by exploiting an underlying model of the phenomenon [24]. Varying levels of coordination and data sharing among robots are coupled with these techniques. For these methods to work, reliance on prior data, on a model, or on navigation strategies that choose between predefined targets is often necessary, highlighting the difficulty of deploying strategies that only exploit sensed input data. In gas sensing applications, prior data are rarely available, and the stochastic and time-variant nature of gas dispersion makes it hard and environment-dependent to model. Moreover, the scarce and punctual nature of data in gas sensing missions makes it difficult to employ non-myopic approaches, even when using an MRS. All these factors contribute to the difficulty of providing the navigation algorithm with additional information beyond the sensed input data.

The application of MRSs to the GDM domain has rarely been explored in the literature. An MRS composed of UAVs was deployed for a mapping mission in [25], in which an estimation method was proposed, based on Gaussian kernels and coupled to a non-adaptive navigation strategy, which consisted of a preplanned lawnmower movement in 2D, where one third of the area was allocated to each of the three drones. In [26], the authors showed that IPP multi-robot strategies for GDM outperformed random walk navigation. However, the method was constrained to 2D, and the robots moved very slowly, at 2 m/min. MRSs have more frequently been employed in the GSL domain. The performance of source localization algorithms was enhanced by coordination among robots in [27]. In [24], a team of robots located a gas source, using IPP planning coupled with a model of the gas distribution. Formation algorithms have also been applied to the domain of multi-robot GSL, for example in [28].

### 1.3. Robotic Gas Sensing and Propeller Interference

To understand the inherent difficulties of robotic gas sensing missions, it is important to become acquainted with the underlying gas plume dispersion. The phenomenon of gas dispersion in the air is characterized by a mixture of the diffusion of the molecules that move away from the source, even when no airflow is present, and the advection of particles due to external airflow [29]. A wind field causes the gas to create a trail, which characterizes the plume dispersion in most real environments. Laminar wind causes the plume to become wider and less concentrated away from the source, while a turbulent flow does not allow the maintenance of a well-characterized shape [30]. Even though gas dispersion can be modeled using Gaussian distributions [29], this simplification does not capture the patchy nature of the plume filament.

The interference of the drone’s propellers has been studied in the context of the impact that it can have on the chemical composition of the surrounding environment, particularly in relation to the optimal gas sensor placement. In [31], Neumann et al. used the airflow of the propellers to convey gas particles to the sensors placed below them. This solution was identified as the best trade-off between a long carbon fiber tube that would avoid turbulence entirely and placing the sensor directly at the bottom of the drone. In [32], particle image velocimetry was used, to visualize the airflow around a quadcopter, with the aim of validating the bespoke sensor configuration. One interesting consideration is that the authors suggested that placing a chemical sensor where the wind speed is highest may not be beneficial, as the sensor’s heater cools down and does not react to gas very well. In our previous work [33], we also assessed the sensing outcome of two sensor placements, concluding that placing the gas sensor at the bottom of the drone yielded better performance.

**Figure 1:**
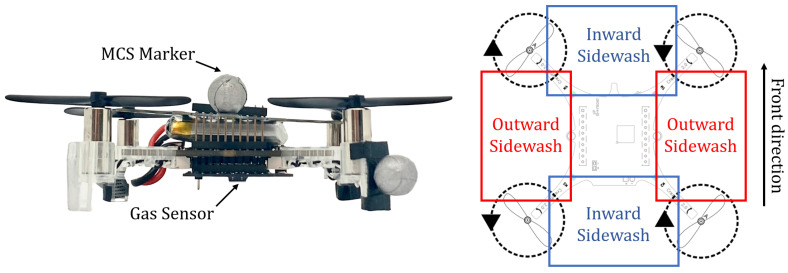
On the left, a picture of the Crazyflie robot used for this work. The gas sensor can be seen at the bottom of the quadrotor. On the right, the propeller configuration of the Crazyflie robot with the corresponding sidewash areas: outward sidewash in red; inward sidewash in blue. CF image taken from bitcraze.io, accessed on 30 August 2023.

More in-depth studies on the profile of the interference caused by the drone’s propellers and its impact on the surrounding environment have focused on assessing the possible, and potentially hazardous, disruption that such an effect can have on the motion of neighboring vehicles. We consider the literature that studied the wake of the propellers in a configuration equal to that of the platform that we selected for this work, which is the Crazyflie V2.1 robot (CF, Bitcraze AB, Malmö, Sweden). A picture of the CF robot, together with a schematic of its propeller configuration, is reported in Figure 1. Additional details about this platform will be given in Section 4.1. A study carried out in [34] explained that counter-rotating propellers generate slipstreams that reinforce or weaken one other when they meet. In a quadrotor propeller configuration equal to that of the CF, the slipstreams are reinforced on the sides of the vehicle and weakened at the front or back of the vehicle. A strong downwash effect is also measured below the central axis of the drone. The work reported in [35] additionally confirmed the presence of the slipstreams, which generate an outward sidewash from the side propellers and an inward sidewash from the front and back propellers. This work also reported measuring a strong downwash effect, which impacted an area much greater than the sidewashes. A visualization of the areas where the inward and outward sidewashes originate from, with the propeller configuration considered in our work, is reported on the right side of Figure 1. The work presented in [36] provided a visualization of the downwash effect of a single robot on a smoke plume.

The presence of the downwash effect was taken into account in [37], where downwash-aware trajectories were designed for a swarm of CF robots, to avoid collisions. The authors placed a virtual ellipsoid around each agent and used it to carry out collision avoidance maneuvers. The dimension of the ellipsoid corresponding to the arrow pointing downwards from the CF’s body was significantly larger than the other two. This prevented each robot from transiting above or below another agent and improved the flying stability of the whole system. The work reported in [38] proposed an algorithm to account for the sidewash and downwash effects that the propellers of a quadrotor have on a gas plume dispersion. The paper presented an algorithm that used a wake model to estimate the real position of the detected gas particles and eliminated the effect of the sidewash and downwash, accordingly. Unfortunately, the lack of details concerning the parametric choices and calculations and the lack of an in-depth experimental evaluation did not allow the work to be extended to other platforms.

### 1.4. Paper Contribution

In our previous work [39], we designed and compared several algorithms for multi-robot gas mapping with different levels of coordination, information sharing between robots and prior environmental knowledge. The results of the paper underlined the benefit of collaboration between robots and the crucial role that sharing information plays in data-scarce applications, like gas mapping. However, the algorithms were only validated in a simulation environment, which did not take into account the effect of the propellers on the gas dispersion, and the interference that the drones can cause on one other. For this paper, we wished to investigate this interference and to deploy some of the algorithms presented in [39] on real multi-robot sensing missions. The contributions of the paper are:

- A qualitative analysis of the interference that a drone has on a gas plume and on a fellow robot, by means of visualizations using a smoke machine;
- A quantitative analysis of the interference, by means of static hovering and plume traversing experiments;
- The deployment of some of the most interesting methods presented in [39] on an MRS flying inside a wind tunnel: namely, the Individualist strategy, the Clustering + *Replan* strategy and the GaSLAM strategy;
- A strategy aimed at reducing drone-on-drone interference, by means of the inclusion of an off-limits interference volume around each robot, and the analysis of its impact on MRS algorithms for GDM and GSL;
- The comparison of our approaches to three baselines: multi-robot preplanned trajectory, single-robot model-free navigation with clusters and single-robot model-based navigation.

For all the approaches, the 3D Kernel DM+V/W was chosen to estimate the gas distribution, because of its light computational requirements. The gas mapping mission was conducted by NAVs moving continuously, to maximize the amount of information gathered within the time constraints.

## 2. Interference Analysis

In this section, we investigate the interference that two drones cause on one other. Initially, we present qualitative results obtained through the visualization of the interference when the drones hovered inside a plume created by a smoke machine. Subsequently, we present a quantitative interference analysis obtained through several static hovering experiments and through plume traversing experiments. Throughout this work, a generic Crazyflie drone will be referred to as CF, while the two drones used experimentally will be referred to as CF1 and CF2.

### 2.1. Smoke Machine Interference Analysis

As discussed in Section 1, the complex and patchy nature of gas dispersion makes it hard to model, even in controlled environmental conditions. The deployment of one or more drones in an area with a gas dispersion can greatly disrupt the plume with the sidewash and downwash effects generated by the propellers. A smoke machine is a useful tool for visualizing a gas plume and, as in our case, witnessing how the drones interact with it. For the purpose of this paper, we used an Aerotech smoke generator placed inside a wind tunnel, with the wind kept constant at 0.7 m/s.

**Figure 2:**
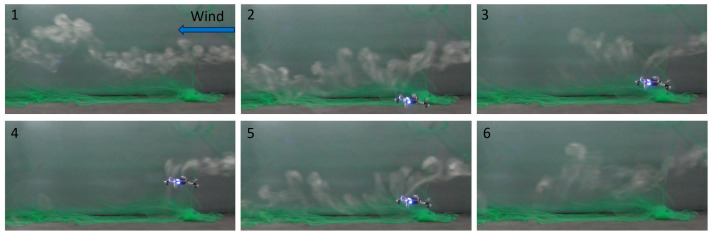
Side view of one CF hovering inside a smoke plume at different time steps: (1) intact smoke plume; (2) CF taking off; (3) beginning of downwashing effect; (4) hovering in the plume; (5) beginning of landing maneuver; (6) first frame where CF not visible.

The patchy nature of the smoke plume, even without drone disturbance, can be seen in the top left corner of Figure 2. Additionally, Figure 2 shows the impact of one CF taking off and hovering inside the smoke plume at different time steps. We can clearly see that, as the drone took off and reached the altitude of the smoke machine’s inlet, smoke particles were pulled into the downwash vortex created by the drone’s propellers. The effect is particularly visible in snippet 4, which was taken during a static hovering motion. The visual experiments highlight the disruption caused by the downwash effect, but this disruption does not necessarily prevent sensing, as the gas particles are conveyed on the sensor placed at the bottom of the drone. This consideration is corroborated by the findings presented in [33], which argued that placing the sensor at the bottom of the body of a CF drone yields a better sensing performance. Another interesting conclusion that can be drawn from snippet 6 of Figure 2 is that, in the presence of wind, the plume recovers its shape relatively quickly after the drone lands. This consideration is especially important in the scope of a multi-robot gas sensing mission where, if plume disruption persists for a very long time, the drones will probably not be able to coexist at a relatively short distance from one other.

**Figure 3:**
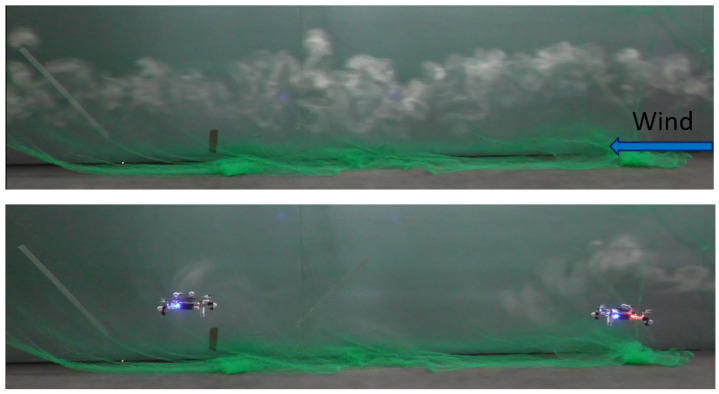
Top figure: side view of the intact smoke plume. Bottom figure: side view of two CFs hovering inside a smoke plume, one meter from one other.

Given the objective, to deploy a multi-robot gas sensing mission, we decided to visualize the effect of two drones hovering inside the smoke plume. The bottom part of Figure 3 shows a side view of two drones hovering in the plume: the drone to the right is 1 m in front of the smoke inlet, and the drone to the left is 2 m in front of the inlet. There is a stark contrast between this image and the top part of Figure 3, which shows the intact smoke plume, highlighting the severity of the drones’ impact. Interestingly, we can see that both drones—even the one to the left, which is further away from the inlet—are surrounded by smoke particles, suggesting that the downwash effect may not push all smoke particles downwards and that sensing for the CF located further away from the gas inlet may still be possible.

**Figure 4:**
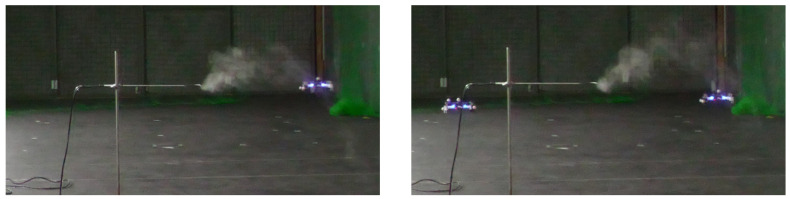
Left figure: front view of one CF hovering inside a smoke plume. Right figure: front view of one CF hovering inside a smoke plume while a second CF hovers 1 m to its left.

To complete the visual analysis of the impact of the drones on the smoke plume, we show a drone hovering inside the smoke plume, with a second drone hovering 1 m next to it, but outside of the plume, in Figure 4. This figure is harder to visualize than the side view. On the left, where only one CF is hovering inside the plume, we can observe a pull of the smoke particles towards the drone, pertaining to the downwashing effect already visualized in Figure 2. When the second drone is added, we can see that the plume is dispersed and pushed slightly upwards, suggesting that the outward sidestream mentioned in [35] might affect sensing.

The images shown in this section are exemplary of behaviors observed during several experimental runs. They are meant to give the reader a qualitative idea about the impact of a drone flying inside a smoke plume but they do not allow for drawing any quantitative conclusions.

### 2.2. Experimental Interference Analysis

The preliminary smoke machine experiments visualized the disruption of the plume caused by one and two CFs. As our research interest concerned gas dispersion, and smoke and ethanol do not necessarily have the same fluid dynamic dispersion properties, we decided to conduct more in-depth experiments, to understand the interference phenomenon on an invisible ethanol plume.

**Figure 5:**
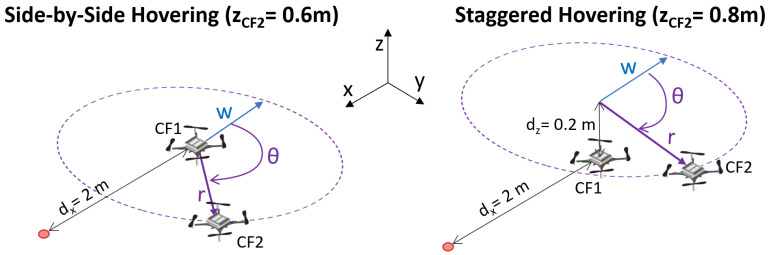
Schematic view of the experimental setup used for interference analysis during the side-by-side and staggered hovering experiments. The CF images are taken from bitcraze.io, accessed on 30 August 2023.

We conducted several experiments, to assess the interference that a quadrotor has on the sensing capabilities of another quadrotor placed inside the gas plume. The experimental setup consisted of a first NAV, CF1, placed 2 m in front of the gas inlet, in the downwind direction. The inlet was positioned 60 cm above the ground, with CF1 hovering in front of it at the same height. We then let another NAV, CF2, hover at different locations around CF1. The hovering positions of CF2 were identified by *r*, the distance vector between CF1 and CF2, and by θ, the angle between the wind direction vector *w* and *r*. We repeated three experiments for each combination of θ and *r*. Additionally, while we always kept the position of CF1 fixed, we evaluated two different height configurations for CF2. In the first one, called *side-by-side* hovering, CF2 was also hovering at 0.6 m. In the second one, called *staggered* hovering, CF2 was hovering at 0.8 m of height. A schematic view of the experimental setup can be seen in Figure 5. In this figure, the side-by-side and staggered hovering configurations are highlighted, as well as an example of *r* and θ pairing, with respect to the wind direction vector *w*. The gas source inlet is represented by a red dot.

Through preliminary experiments, we established a threshold, to distinguish gas sensing values that were definitely part of the plume from those that could be considered as weaker readings or background readings. The objective of this threshold was to let us see if the sensing performance of CF1 was impacted by the presence of CF2. For simplicity, we will refer to the binary results identified by the threshold as gaseous and non-gaseous. We considered two different experimental phases: the initial phase, where CF1 was hovering in the plume and CF2 was on the ground, and the interference phase, where CF2 was hovering next to CF1. For each phase, we could compute the percentage of data that was considered as gaseous, using the threshold, and evaluate the change in sensing between the two phases. In each phase, we collected gas readings for 20 s at 10 Hz.

We first gathered the baseline gas sensing data, with only CF1 taking off and hovering in the plume, which resulted in 75% of the gaseous data sensed. This result once again reinforced the findings of our previous work, in particular [33], suggesting that our setup allows good sensing performance, despite the propeller impact.

**Figure 6:**
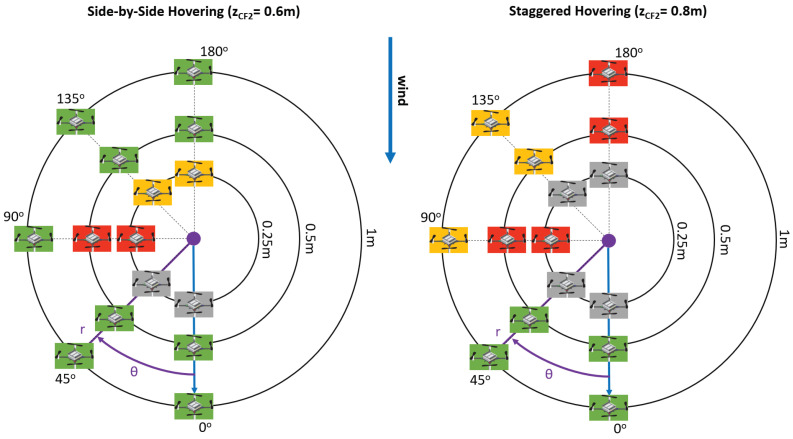
Visualization of the interference effect of CF2 on the sensing performance of CF1. Side-by-side hovering is on the left, staggered hovering is on the right. Blue arrows represent the wind direction. An example of *r* and θ is also shown. CF images are taken from bitcraze.io, accessed on 30 August 2023.

The results of the side-by-side hovering experiments are visualized on the left side of Figure 6, while the staggered hovering results are reported on the right side. The quantitative results of all the experiments can be found in the Appendix A. We opted for a commentary on a simpler visualization rather than on raw data to ease the reader. The visualization features experiments only on half circles around CF1, because the effects are mirrored on the other half. The purple dot in Figure 6 represents the position of CF1, while the drone images represent the positions of CF2 around CF1. The reader can also notice the blue arrows, identifying the wind direction, and an example of *r* and θ pairing.

Figure 6 uses a color scheme to visually represent the effect that each configuration of CF2 had on the sensing performance of CF1. We used the color orange to highlight a sensing percentage decrease between 30 and 60%, which we considered as moderate, while the color red was used to highlight sensing percentage decreases greater than 60%, which were considered severe. The color green was used to identify a sensing percentage decrease lower than 30%, which we considered negligible. We chose a rather high threshold for negligible interference, because of the stochastic nature of plume dispersion and gas sensing, preferring to focus on results that strongly underlined the presence of interference. Finally, the color gray was used to indicate configurations in which one or both the drones could not fly (see below for additional explanations). Throughout the commentary of the rest of this section, we will use the terms “upwind” and “downwind” to identify the positions of CF2 with respect to CF1, according to the wind direction. Concretely, the downwind configuration happened at θ equal to $0^{\circ }$ and $45^{\circ }$, while the upwind one happened at θ equal to $135^{\circ }$ and $180^{\circ }$.

The visualization on the left side of Figure 6 shows that, in the side-by-side hovering experiment, when CF2 was located downwind from CF1, there was little-to-no sensing percentage decrease. When CF2 was located upwind, we observed a moderate interference effect when the drones were very close to one other. This finding coincided with our visual analysis reported in Figure 3. We think that the wind direction also played a role in ensuring that the sensing performance was not very degraded when CF2 was located upwind. Finally, when CF2 was next to CF1 ($\theta =90^{\circ }$), we observed a significant interference effect on the sensing performance. We can attribute this effect to the outward sidewash. It is interesting to note that the inward sidewash, which should be present at $\theta =0^{\circ }$ and $180^{\circ }$, was not as pronounced. This confirms the findings reported in [34,35], which underlined the stronger nature of the outward sidewash compared to the inward one.

The visualization on the right side of Figure 6, pertaining to the staggered hovering experiments, shows that, when CF2 was hovering above CF1, the interference effect was more severe overall and predominantly present when CF2 was upwind or to the side of CF1. When CF2 was upwind, we could attribute the interference to the downwash effect of the propellers, which prevented gas particles from reaching CF1. A similar effect may have been happening when CF2 was placed on the side, although *r* was quite large in this setup and we were not expecting to see such an impact. Further investigation should be dedicated to clarifying this point.

The gray boxes in Figure 6 indicate a configuration where one or both CFs could not fly. This happened for two different reasons. When θ equaled $0^{\circ }$ and $45^{\circ }$, CF2 could not take off, because of the downwash effect generated by CF1, which was already in the air. This happened only when CF2 was downwind, because the downwash effect of CF1 was pushed towards CF2 by the wind. Similarly, in the staggered configuration, the takeoff motion of CF2 caused CF1 to crash. This happened when CF2 was upwind, causing its downwash effect to be pushed towards CF1 by the external wind. These findings add to the work presented in [37], which highlighted the urgency of planning downwash-aware trajectories for CF swarms.

To conclude our interference analysis, we carried out experiments where CF1 traversed the length of the gas plume by flying towards the gas inlet for 2.5 m, with CF2 flying next to it. We decided to evaluate the most interesting configurations, according to the static hovering interference analysis. Namely, we kept CF2 at r=0.5 m from CF1 and at angles $90^{\circ }$ and $180^{\circ }$, and we explored both side-by-side and staggered configurations.

In this set of experiments, the interference phase was intended as the entirety of a flight of CF1 towards the plume inlet, with CF2 next to it. The baseline experiments, where CF1 traversed the plume without CF2 flying next to it, yielded a sensing percentage of 71%, on average. Table 1 shows the relative change between the normalized number of samples above the threshold during the interference phase and the baseline experiments. The color code is the same as Figure 6. The results presented in Table 1 show that, in motion and at $\theta =90^{\circ }$, the strongest interference was detected in the side-by-side configuration, caused by the outward sidewash. At $\theta =180^{\circ }$, the strongest interference was in the staggered configuration, caused by the downwash. These results were aligned with the experimental findings of the static hovering and are additionally supported by the literature in this field [34,35].

The first conclusion we can draw from our experimental campaign, supported by the baseline experiments, is that a single CF that hovers in or traverses a gas plume can detect the presence of gas quite reliably. Additionally, we can conclude that the gas sensing performance of a CF drone is impacted when a second drone flies next to it in specific configurations. In particular, the interference is prominent when CF2 was next to CF1 and when CF2 was flying above and in front of CF1.

## 3. Multi-Robot Gas Sensing Algorithms

In this section, we present gas sensing algorithms for a multi-robot system composed of two CFs. These methods were first presented in our previous work [39], where we focused on the design of such algorithms and tested them in simulation. In this paper, we additionally propose a method to take the drone interference into account, and we deployed the algorithms in a real testbed. We present two model-free navigation strategies with varying degrees of coordination (Individualist and Collaborative with Clustering) and one model-based strategy for simultaneous GDM and GSL (GaSLAM), which we compared to three baselines. Each of the strategies employed the same gas mapping estimation method, although the model-based strategy benefited from additional interpolation with the final model-based map. The model-free approaches followed the same IPP navigation strategy, while the navigation strategy for the model-based approach was also based on source location guesses.

For the purpose of tractability, the experimental volume was divided into *N* cells, of size 10×10×10 cm^3^. The methods are presented in a generalized way for a system of *k* robots.

### 3.1. Gas Map Estimation

The estimation of the gas distribution in the explored volume was achieved using the 3D Kernel DM+V/W algorithm, presented in [8]. This algorithm builds a 3D map of the gas dispersion, by weighting the collected gas samples with a multivariate Gaussian function. The weight of each new sample for each of the *N* cells is found by evaluating a Gaussian kernel at the distance between the location of the measurement and the center of the cell. The shape of the kernel is controlled by the covariance matrix and depends on the kernel width, which encodes the amount of extrapolation from individual readings and from wind information. We selected the parameters of this algorithm in accordance with previous work [4,17]. The wind information, retrieved, for example, with an onboard anemometer, could also be included in the estimation. However, for the scope of this work, we assumed that the wind intensity and direction were constant and known throughout each experiment.

### 3.2. Navigation

The navigation strategy for model-free approaches uses an adaptive IPP algorithm to select the next goal positions. The informative quantity used by the IPP algorithm is the Kullback–Leibler Divergence (KLD) [40], and it is used to maximize the information gathered during navigation. This quantity was successfully used in our previous work on GDM and GDM+GSL (GaSLAM) [4,17]. The KLD quantifies the difference between two probability distributions and is computed as

(1) $$D_{KL}\left(P||Q\right)=\sum_{i}P\left(i\right)\log_{2}\left(\frac{P(i)}{Q(i)}\right),$$

where *P* is the current probability distribution of the gas samples in each cell, and *Q* is the estimated next step probability distribution, obtained by adding one virtual sample, corresponding to the expected value of the distribution, to *P*. A higher value of KLD indicates discrepancies between *P* and *Q* and suggests that more exploration is needed, to increase the confidence of the gas estimation in a cell.

### 3.3. Coordination Strategies

An MRS can present several levels of coordination among its members. In our previous work [39], we compared several strategies with varying degrees of coordination, for a simulated multi-robot system composed of two drones. For this paper, we evaluated some of these strategies in a real experimental setting. The rest of this section will outline the strategies that were deployed on real drones for this paper.

#### 3.3.1. Individualist Strategy

Employing the Individualist strategy, the robots independently built their own gas distribution map, based on the samples that they acquired on their own within the time limits. The robots could only use their own map to select the next goal positions, using the IPP strategy reported in Section 3.2, making the selection entirely reliant on local estimation. When the time budget elapsed, a final gas map, built by combining the *k* maps produced by the robots, was evaluated. As an estimation of the gas value could be provided by more than one robot for each cell, we selected the gas value with the lowest associated variance. The Individualist strategy yielded the worst performance in simulation, but we chose to evaluate it with real experiments, because it is the easiest strategy to deploy in real-world missions, as it does not require communication among the robots.

#### 3.3.2. Collaborative Strategy with Clustering

Collaborative strategies rely on the robots sharing their gas samples to build a shared gas map, which all the teammates can use to select their next goal positions. Additionally, movement coordination allows the robots to improve the allocation of their goal positions. In this work, we deployed a movement allocation method previously presented in [39], called *Replan*.

In the *Replan* movement allocation strategy, whenever a robot reaches a goal position, it will find *k* possible next goals. The goals are selected with the IPP-based navigation criterion, explained in Section 3.2, and are at a safe distance $d_{s}$ from one other. The Hungarian algorithm is then used to allocate the *k* goals to all the *k* robots in the system, based on the shortest distance covered. All the robots immediately switch to their new goal position. The objective of this strategy is to allow for a robot to replan for itself and all its teammates, based on the information that the robots have acquired since the last replanning. In fact, the new information could offer more insight on where to go next and could guide all the robots to more informative locations.

In our previous work [4], and in the simulation results presented in [39], we showed the benefits of coupling IPP strategies to a spatial clustering method. Consequently, we decided to apply the same method to the *Replan* strategy. The method consists in dividing the volume in *K* clusters, using the K-means algorithm, and exploring all the clusters sequentially. The number of clusters chosen for this work was set to four. As the vehicles in the MRS concurrently operated in the same cluster, we chose to have clusters that were big enough to avoid excessive interference among the drones, and to avoid biasing the mission to achieve high volume coverage, instead of trying to focus primarily on the plume. Although the addition of clusters did not change the reliance on local data only, the system could now count on a more spatially constrained navigation, which pushed for exploration. The cluster method corresponds to a less spatially constrained version of visiting predefined waypoints.

### 3.4. Model-Based Navigation

GaSLAM [17] is an algorithm for simultaneous gas mapping and source localization. It organically combines two State-of-the-Art methods—3D Kernel DM+V/W for mapping and Source Term Estimation (STE) [41] for localization—to produce a reliable gas map and to localize the gas source within it. The sensed gas data is interpolated, using the 3D Kernel DM+V/W algorithm, and the resulting map is used by the STE to create a belief about the source location. As STE is a model-based algorithm, we effectively have two maps of the environment at all times: one is the map generated by the Kernel algorithm (the Kernel Map), which only contains locally sensed data, and one is the map given by the STE model (the STE Map), which uses the belief about the STE parameters to guess the gas distribution in the whole environment. At the end of a run, the two maps are combined, by integrating the STE Map with the readings perceived as gaseous from the Kernel Map.

Navigation is vector-based. In the initial exploratory phase, the navigation vector is the sum of a vector pointing to the highest KLD and a vector pointing to the belief about the source. The following exploitative phase, which starts when the belief about the source location position is deemed good enough, focuses on exploring areas where the difference between the Kernel Map and the STE Map is high. For more details, see [17].

In this paper, we coupled the *Replan* movement coordination strategy to an MRS running GaSLAM, to observe the effects of the injection of global knowledge into the system. Global knowledge comes from the gas dispersion model used by the STE, which, in this case, was the pseudo-Gaussian model [42].

In addition to a gas map of the concerned volume, GaSLAM also provided a source localization guess. We will also analyze the accuracy of this guess.

### 3.5. Baselines

Three baseline methods were used in the evaluation of the multi-robot sensing algorithms proposed. The first one was the lawnmower method, which is a non-adaptive path planning strategy consisting of a zig-zag motion. The robots were kept at a distance equal to $r_{co}$ from one other during the scans, where $r_{co}$ was the update radius of the 3D Kernel DM+V/W algorithm. Concretely, this lawnmower motion kept the robots far enough apart so that they would not update the same cells, therefore maximizing the coverage property, but close enough that interference among the robots might impact the performance.

The second baseline was a single-robot method, where navigation uses IPP and clusters, taken from [4]. The final baseline consisted of a single robot running the model-based GaSLAM algorithm [17]. For the single robot baselines, the total flying time was set to twice the time of the experiments involving two robots. This was done deliberately, to compare the mission outcomes in single- and multi-robot systems where the total flight (and therefore sensing) time was the same, independently of the size of the MRS. However, MRS missions could be conducted in less run time, as the time budget was split between the robots.

### 3.6. Interference Volume and Collision Avoidance

The smoke machine and interference experiments presented in Section 2 highlighted the presence of interference between drones during a gas sensing task. However, the interference experiments did not assess whether this interference impacted gas sensing missions or if it was necessary to design algorithms that would take it into account.

According to the conclusions of Section 2, the interference was prominent when the drones were next to one other or when one was behind and below the other. In order to prevent the drones from being impacted in their sensing mission, we modeled the interference effect as an Interference Volume (IV) shaped like a rectangular cuboid. The IV stretched on both sides of the drone, below it and behind it.

**Figure 7:**
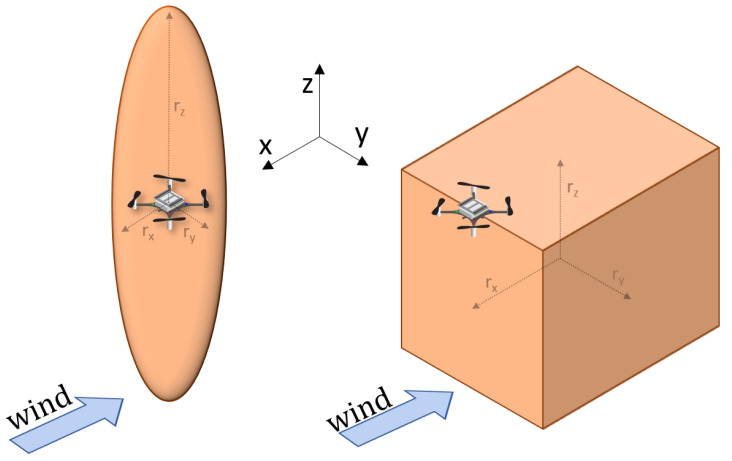
Ellipsoid collision avoidance ($r_{x}$ = $r_{y}$ = 0.15 m, $r_{z}$ = 1.8 m) and IV collision avoidance ($r_{x}$ = $r_{z}$ = 0.5 m, $r_{y}$ = 0.25 m). No interpenetration of the collision avoidance volumes was allowed. The sensing was virtual, carried out through Motion Capture System data.

We took inspiration from the work of [37], where a virtual ellipsoid was placed around each vehicle. The ellipsoid established the volume for Collision Avoidance (CA) maneuvers, preventing each robot from transiting above or below another team member and improving the flying stability of the whole system. The dimension of the ellipsoid corresponding to the arrow pointing downwards from the CF’s body was significantly larger than the other two. A schematic view of the ellipsoid can be seen on the left side of Figure 7. The smaller radii of the ellipsoid, $r_{x}=r_{y}$, were kept at 0.15 m. As CA was enabled for all robots, the robots were not able to get closer than 0.3 m to one other. We selected this distance to prevent flight accidents, similar to the ones described in Figure 6. We selected a very large $r_{z}$ for the ellipsoid, to forbid the drones from flying below one other in our wind tunnel setting.

For this paper, we proposed to substitute the ellipsoid with the IV defined above, to allow for CA that aimed to prevent interference in gas sensing. The positioning of the IV with respect to the CF can be seen on the right side of Figure 7. It was shifted behind and below the CF, according to the conclusions drawn from Section 2. The dimensions were chosen according to the experiments reported in Figure 6. It must be noted that, as CA was enabled for all robots, the drones could not get closer than 0.5 m to one another on the Y axis, even though $r_{y}$ was set to 0.25 m. In the practical implementation of the IV, the rectangular cuboid was fully encapsulated inside an ellipsoid, in order to make CA maneuvers smoother. In case a deadlock occurred, due to the large nature of the IV, we opted to give moving priority to CF1. In the MRS algorithms analyzed in this paper, the ellipsoid CA was maintained wherever it was not explicitly stated that the IV was used.

## 4. Performance Evaluation

In this section, we present the experimental platform and the experimental testbed used in this work. We then explain how the ground truth map was gathered and how the gas sensors were calibrated. Finally, we present the metrics that were used to evaluate the performance of the gas sensing algorithms.

### 4.1. Experimental Platform

The platform chosen for this work was the Crazyflie V2.1 robot, successfully used for gas detection tasks in our previous work [4,17,33]. The CF had a diameter of 10 cm, and its propellers had a radius equal to 2 cm. It was able to carry a payload of up to 15 g and could fly to up to 7 min without payload. The CFs were equipped with a custom Printable Circuit Board (PCB) featuring a MiCS-5524 gas sensor (SGX Sensortech, Neuchatel, Switzerland). This sensor was chosen because of its small form factor, which allowed for it to be easily embedded in a CF, and for its fast reaction and recovery time, observed during preliminary tests. The data were converted from analog to digital by an ultralow power STM8 controller, which sampled the data at 10 Hz. Localization was achieved with the help of a Motion Capture System (MCS) with millimetric accuracy.

The architecture of the robotic system was centralized, with computation performed on a PC, which acted as a base station. Localization data were broadcast to all CFs, in order to allow for collision avoidance. The overall software architecture of the system was adapted from the Crazyswarm project [43].

### 4.2. Experimental Testbed

The experiments were carried out in a wind tunnel of volume 18 × 4 × 2 m^3^. Inside the tunnel, the wind speed could be adjusted and was laminarized by a dedicated honeycomb filter. The volume of the tunnel effectively used for the experiments was 7 × 2 × 0.5 m^3^. Constraints on the volume were caused by the limited flight time of the experiments. Given the size and capabilities of the drone, this volume was comparable to the ones previously used in the literature for GDM. The wind speed was kept at 0.7 m/s throughout all the experiments. Limitations in the testing regime employed in this paper could yield different results from experiments done in environments with higher turbulence or different gas dispersion rates.

The gas used during the experiments was ethanol, released by a stationary electric pumping device. The source release rate was set to 0.5 L/min, and corresponded to a mix of air and ethanol released by the pump. The environmental conditions chosen for this work were the ones that provided the best sensing results in the 3D GSL experiments presented in [33]. The initial position of the robot was the same for all approaches and corresponded to the right corner of the volume opposite the source. For each MRS strategy, five experiments were carried out, each lasting 2 min and 15 s. The baseline experiments featuring single-robot strategies lasted 4 min and 30 s.

### 4.3. Ground Truth and Sensor Calibration

The ground truth data were acquired using an array of static sensors mounted on a traversing system (a three-axis robotic manipulator present in many wind tunnels). The sensors sampled at 10 Hz for a duration of 2 s at the center of each grid cell. After eliminating the outliers, the data obtained were averaged, to produce the ground truth.

Sensor calibration was necessary, due to the small manufacturing differences introduced by each sensor. A linear regression model was used, to describe the relationship between the data coming from each static sensor and the data coming from the CF’s sensors, while moving on a predefined path that traversed the plume.

### 4.4. Evaluation Metrics

Two metrics were used, to quantify the performance of the gas mapping strategies presented in this paper: coverage and shape coverage.

The coverage metric evaluated how much exploration was accomplished by each strategy. It was defined as the number of cells $N_{u}$ whose gas value had been updated over the total number of cells *N*:

(2) $$C=\frac{N_{u}}{N}.$$

The shape coverage metric, which represented the probability of an updated cell being identified correctly as containing gas or not, according to the ground truth, was computed as

(3) $$SC=p(d_{i}\geq th_{d}|g_{i}\geq th_{g}\left|\right|d_{i}<th_{d}\left|g_{i}<th_{g}\right)\;\forall i\in N_{u},$$

where $d_{i}$ was the obtained gas value of each grid cell, $g_{i}$ was the corresponding ground truth value, $th_{d}$ and $th_{g}$ were the thresholds used to determine whether or not a cell contained gas and $N_{u}$ was the number of updated cells. The ground truth threshold $th_{g}$ could easily be determined by the data and prior knowledge of the shape of the plume. The data threshold $th_{d}$ was harder to determine. The sensitivity of the sensors varied significantly, due to environmental factors, and the stochastic nature of the gas dispersion coupled with the propeller perturbation made it hard to establish a threshold that was valid for all runs. We therefore opted to employ a dynamic thresholding algorithm, found in [44].

We intentionally opted out of using the Root Mean Square Error (RMSE) to compare the gas maps to one other, because of the fact that our datasets were taken on different days, with variations of environmental conditions, such as temperature and humidity, that could affect the performance of the gas sensor. Opting for a binary division of the map through the shape coverage metric allowed us to carry out a fairer comparison between different strategies.

The performance of the gas source localization guesses obtained through the GaSLAM algorithm was evaluated, by computing the error between the source guess and the true source position across all three axes.

## 5. Results

In this section, we present the results of the experimental validation of the algorithms described in Section 3 using a multi-robot system composed of two CFs flying inside a wind tunnel with constant wind.

**Figure 8:**
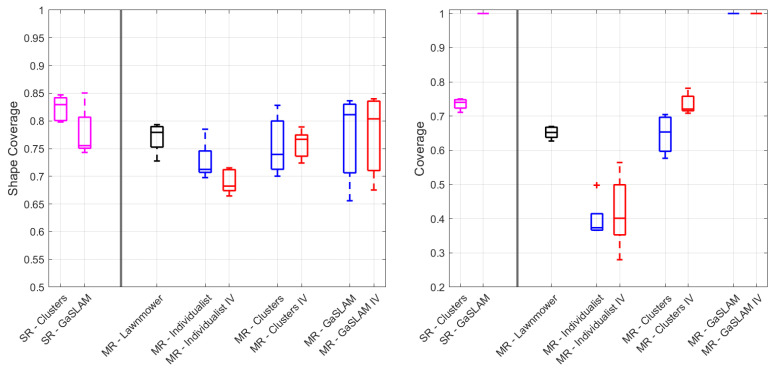
Shape coverage and coverage metrics for all algorithms presented. The single robots (SR) baselines are shown in magenta, while the multi-robot (MR) lawnmower is shown in black. The blue and red boxes are used for the different MR gas mapping algorithms, without and with the IV, respectively.

Figure 8 shows the gas mapping metrics for the algorithms evaluated in this paper. The single robots baselines are shown in magenta, while the multi-robot lawnmower is shown in black. The blue and red boxes are used for the different gas mapping algorithms, without and with the IV, respectively. All types of algorithms were evaluated by running them five times.

The Individualist strategy yielded the worst performances, both in terms of shape coverage and of coverage. Interestingly, the addition of the IV impacted negatively the shape coverage metric, but introduced a slight benefit in the coverage metric. This can be explained by the fact that when the robots had to respect the larger collision avoidance constraints imposed by the IV, they were forced to explore more. However, when this was coupled with the Individualist strategy, which did not prompt the robots to explore the environment much, the robots performed collision avoidance more often. When doing so, the robots accelerated, and their sensing performance degraded as a consequence, impacting the shape coverage metric.

We can see a slight improvement in the shape coverage performance in the multi-robot Clustering strategy using the IV, coupled with a significant improvement in the coverage metric. While the coverage metric improvement can be attributed to the fact that the robots were kept further apart and forced to explore more, the slight improvement in the shape coverage metric suggests that the robots were less susceptible to interference with one other. This was reinforced by the coupling of the two metrics, indicating that the robots were able to explore a larger area while producing an accurate map.

The GaSLAM runs always provided perfect coverage of the environment, in virtue of the interpolation with the gas map provided by the model-based GSL strategy. The shape coverage metric was, on average, the highest among the multi-robot methods, but suffered from quite a large variance. This was due to the fact that, when the GSL algorithm converged to a source location value that was not very close to the true source position, the consequent gas distribution model was offset, with respect to the true distribution.

The lawnmower movement achieved good performance in both metrics, but it was not the best-performing strategy overall. For gas mapping missions, the lawnmower remains a solid baseline approach that is unfortunately not very scalable. Some interesting conclusions can be drawn, by looking at the difference between the single-robot runs and their multi-robot counterpart. For the Clustering method, we can see that the single-robot run performed equally to the multi-robot IV strategy, in terms of coverage, but that it presented a significant increase in performance, in terms of shape coverage, which described the quality of the map. Here is where we can clearly observe the effects caused by the interference, which impacted the multi-robot strategy, even though the same volume coverage was achieved. The comparison between single-robot and multi-robot GaSLAM highlights an improvement in the shape coverage metric for the multi-robot strategies. This can be explained by the fact that simultaneously gathering more data in the volume can help the model converge faster to a good enough source estimation guess.

Overall, we must conclude that the introduction of the IV only yielded improvements in the multi-robot Clustering strategy. This was somewhat contradictory to what we observed in the interference analysis presented in Section 2. This can be explained by several factors. First, our interference analysis experiments focused primarily on a hovering motion of the CFs. The hovering motion subjected the drones to the prolonged effect of the propeller dispersion and corresponded to a worst case scenario, with respect to movements carried out by the algorithms we proposed. Additionally, the interference we detected in Section 2.2 can be understood as a significant loss of sensing capabilities, but this loss could still allow for correct binary classification of gaseous and non-gaseous zones, according to the shape coverage metric. Finally, the usage of an interpolation method, such as the 3D Kernel DM+V/W, could help mitigate the effect of the interference on the final map.

**Figure 9:**
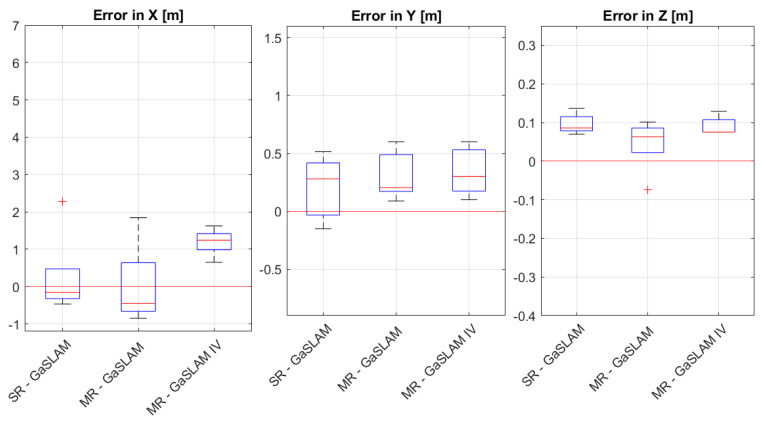
Source localization error obtained through the GaSLAM algorithm across the three axes.

The gas source localization error obtained through the GaSLAM algorithm for single- and multi-robot strategies is shown in Figure 9. We can observe that there was no significant difference in error in Y and Z across the different algorithms. In X, we can see that the best-performing strategy was the single-robot one, closely followed by the multi-robot one, without IV. The multi-robot strategy with IV suffered from a rather large error in X. X, the axis parallel to the wind flow, was the hardest to estimate, and this error seems to indicate that the robots were not able to position themselves close to the source, to refine their guess. This may have been partially due to the imposition of a large collision avoidance constraint through the IV.

The fact that there was no large difference between the source localization performance in single-robot versus multi-robot strategies, makes for a compelling case for the exploitation of MRSs in applications where time is particularly sensitive. In fact, the MRS was able to achieve a good gas localization performance in half the time, compared to the single-robot equivalent.

**Figure 10:**
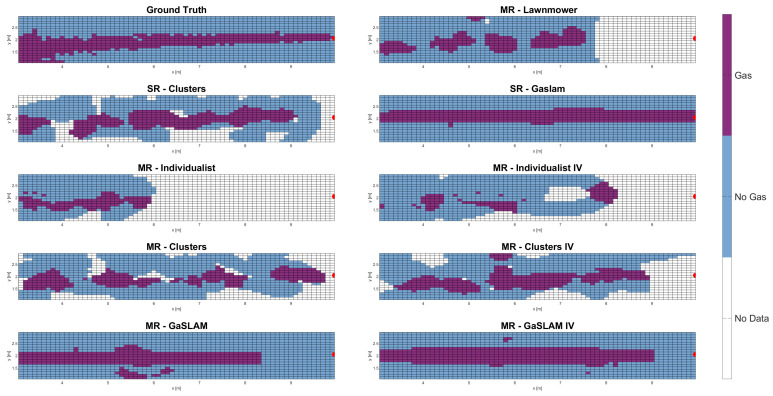
Final gas maps obtained through the strategies presented in this paper. Note that the Z-axis is omitted, to simplify the visualization, and that only the map directly in front of the gas plume is portrayed. The red dot represents the true gas source location and the wind blows from right to left. The coordinate origin is in a corner of the wind tunnel, which is reflected by the fact that the X-axis and the Y-axis do not start from 0 in the figure.

Examples of a slice of the final gas map obtained through the strategies explored in this paper are presented in Figure 10. The ground truth is shown in the top left corner of the figure. The multi-robot lawnmower map highlights the downsides of the lawnmower: namely, no coverage of some part of the map and the fact that the final map can present several chunks of gas rather than a smooth plume. Individualist strategy maps show the non-competitiveness of these strategies, both in terms of poor coverage and patchy gas map reconstruction.

All the cluster-based methods provided good map reconstruction and were able to identify the presence of gas throughout almost the whole length of the plume. The GaSLAM-based methods provided full coverage of the map and, when the gas source location guess was very accurate (as was the case for the single-robot GaSLAM strategy in Figure 10), they provided a final map that most closely resembled the ground truth. Unfortunately, these methods had to rely on a solid gas dispersion model to work—something that is not always easy to obtain for gas applications.

It is interesting to note that several of the multi-robot maps presented small patches of gas, often in areas where gas was not located according to the ground truth. These patches were an effect of the dispersion of gas particles caused by a robot, which could cause the other robots to sense gas where there was none. Fortunately, these patches seem minor and did not impact the overall results.

## 6. Conclusions and Outlook

In this paper, we deployed several algorithms for GDM and GSL on an MRS composed of two CF drones. As propellers can have a significant effect on gas sensing, intensified by the presence of multiple vehicles, we carried out an experimental analysis of the interference that drones cause on one other’s sensing performance, prior to deploying the gas sensing algorithms. The qualitative and quantitative analysis of this interference highlighted degradation in sensing in several flying and hovering configurations. We consequently tested if the addition of an off-limits IV around each drone during GDM and GSL missions helped to mitigate the interference effect.

We concluded that the GDM and GSL algorithms deployed for an MRS during this work performed well, overall. The introduction of the IV was beneficial in the case of the Clustering + *Replan* method, highlighted by an improvement in both metrics. However, the benefit did not translate equally well to the other strategies analyzed, and even caused a reduction in performance in the Individualist strategy. Nonetheless, it is important to highlight the robustness shown by the GDM and GSL algorithms to the presence of documented interference, which opens the doors to further investigations and applications in this domain.

Future work will focus on analyzing environmental and experimental scenarios where the interference could play a bigger role, such as lower or no wind configurations, or MRS featuring a larger number of robots. Finally, attention should be dedicated to addressing the artifacts visualized in the final map reconstructions that highlight the presence of small gas patches in areas of no gas.

## Figures and Tables

**Table 1:** Sensing percentage change between the normalized number of samples above the threshold during the interference phase and the baseline runs in experiments where CF1 traversed the length of the plume and CF2 flew next to it in different configurations. Arrows pointing downwards indicate a decrease in sensing percentage.

Configuration	Angle θ	r [m]	Sensing Percentage Change
Side-by-side	90${}^{\circ }$	0.5	↓ 74.9%
Staggered	90${}^{\circ }$	0.5	↓ 22.2%
Side-by-side	180${}^{\circ }$	0.5	↓ 30.0%
Staggered	180${}^{\circ }$	0.5	↓ 75.2%

## Data Availability

The data presented in this study are available on request from the corresponding author.

## Abbreviations

The following abbreviations are used in this manuscript:

GDM	Gas Distribution Mapping
GSL	Gas Source Localization
MRS	Multi-Robot System
NAV	Nano Aerial Vehicle
IPP	Informative Path Planning
STE	Source-Term Estimation
UAV	Unmanned Aerial Vehicles
CF	Crazyflie
KLD	Kullback–Leibler Divergence
IV	Interference Volume
CA	Collision Avoidance

## Appendix A

Table A1 reports the quantitative results of the side-by-side hovering experiments, consisting of an average of three runs per θ and *r* for the initial and interference phases, together with the sensing difference, expressed in percentage, between the two phases. The columns corresponding to the initial and interference phases report the normalized number of samples above the concentration threshold. A value of 1 indicates that all the samples showed a concentration above the threshold, while a value of 0 indicates that none of them were above the threshold. The last column shows the relative change between the two values, in percentages (e.g., (0.88 − 0.64)/0.88 = 0.275 = 27.5%).

**Table A1:** Normalized number of samples above the concentration threshold during the initial and interference phases of the side-by-side hovering experiments, and percentage change between the two. The color orange was used to highlight a sensing percentage decrease between 30 and 60%, considered as moderate, while the color red was used to highlight sensing percentage decreases greater than 60%, considered severe. Arrows pointing downwards indicate a decrease in sensing percentage, while arrows pointing upwards indicate an increase in sensing percentage.

Angle θ	r [m]	Initial Phase CF1	Interference Phase	Percentage Change
0${}^{\circ }$	0.5	0.88	0.64	↓ 27.5%
0${}^{\circ }$	1	0.92	0.94	↑ 2.0%
45${}^{\circ }$	0.5	0.85	0.87	↑ 1.2%
45${}^{\circ }$	1	0.93	0.99	↑ 6.1%
90${}^{\circ }$	0.25	0.79	0.09	↓ 88.1%
90${}^{\circ }$	0.5	0.84	0.11	↓ 86.5%
90${}^{\circ }$	1	0.61	0.46	↓ 23.9%
135${}^{\circ }$	0.25	0.89	0.58	↓ 35.7%
135${}^{\circ }$	0.5	0.79	1	↑ 26.3%
135${}^{\circ }$	1	0.92	1	↑ 8.4%
180${}^{\circ }$	0.25	0.78	0.54	↓ 30.0%
180${}^{\circ }$	0.5	0.79	0.80	↑ 1.6%
180${}^{\circ }$	1	0.76	0.70	↓ 8.0%

Table A2 reports the quantitative results of the staggered hovering experiments, consisting of an average of three runs per θ and *r* for the initial and interference phases, together with the sensing difference, expressed in percentages, between the two phases.

**Table A2:** Normalized number of samples above the concentration threshold during the initial and interference phases of the staggered hovering experiments, and percentage change between the two. The color orange was used to highlight a sensing percentage decrease between 30 and 60%, considered as moderate, while the color red was used to highlight sensing percentage decreases greater than 60%, considered severe. Arrows pointing downwards indicate a decrease in sensing percentage, while arrows pointing upwards indicate an increase in sensing percentage.

Angle	r [m]	Initial Phase CF1	Interference Phase	Percentage Change
0${}^{\circ }$	0.5	0.91	0.88	↓ 2.7%
0${}^{\circ }$	1	0.84	0.95	↑ 13.7%
45${}^{\circ }$	0.5	0.92	0.89	↓ 2.9%
45${}^{\circ }$	1	0.91	0.76	↓ 16.3%
90${}^{\circ }$	0.25	0.78	0.10	↓ 87.6%
90${}^{\circ }$	0.5	0.82	0.09	↓ 88.9%
90${}^{\circ }$	1	0.84	0.50	↓ 40.2%
135${}^{\circ }$	0.5	0.94	0.44	↓ 53.2%
135${}^{\circ }$	1	0.91	0.62	↓ 31.9%
180${}^{\circ }$	0.5	0.94	0.12	↓ 86.4%
180${}^{\circ }$	1	0.86	0.20	↓ 76.4%
